# Editorial: Hereditary Cancer Risk Assessment: New Perspectives and Challenges for the Next-Gen Sequencing Era

**DOI:** 10.3389/fonc.2016.00133

**Published:** 2016-06-03

**Authors:** Israel Gomy

**Affiliations:** ^1^Institute of Hematology and Oncology, Curitiba, Brazil

**Keywords:** genetic counseling, risk assessment, next-generation sequencing, germline variants, susceptibility, incidental findings, hereditary cancer predisposition syndromes

**The Editorial on the Research Topic**

**Hereditary Cancer Risk Assessment: New Perspectives and Challenges for the Next-Gen Sequencing Era**

One of the greatest challenges in counseling families with cancer is conferring precise information regarding genetic susceptibilities, because it allows a better informed decision-making process about risk management, clinical surveillance, targeted-therapies, and preventive measures.

Genetic susceptibility to cancer can be driven by low-, intermediate-, or high-risk alleles in accordance to penetrance. Low-risk alleles comprise germline variants that are commonly shared among individuals, whose risks are near the risk of the general population, but, when many of them are inherited, they may increase risk substantially. For susceptible carriers, evidence-based guidelines do not yet exist for cancer screening. Intermediate-risk alleles have a moderate penetrance, although there are limited data on the degree of cancer risk, because they may be influenced by gene–gene or gene–environment interactions. For the carriers of these variants, there are no clear guidelines on risk management, and in many cases, the information from testing does not modify clinical management compared to that based on family history alone. Finally, the high-risk alleles are those that cause familial cancer syndromes with high penetrance. For these deleterious variants, genetic counseling and clinical management provide the greatest benefits, but, simultaneously, uncover the greatest challenges in terms of psychosocial and ethical issues.

One such familial cancer syndrome is Li–Fraumeni, which arises from germline mutations in TP53. This syndrome has been extensively studied in the Brazilian population, where a founder mutation is present (1). Nogueira et al. described the use of 18F-FDG-PET/CT for cancer detection in asymptomatic TP53 mutation carriers. Twenty percent of patients showed lesions, supporting the clinical utility of PET/CT in addition to other imaging procedures for screening (Nogueira et al.).

Regarding psychosocial problems with genetic testing, Quinlivan et al. analyzed the attitudes of recent mothers toward a fictitious test for genes conferring a 50% risk of cancer. Only one-third of participants indicated that would have undergone the genetic test. The decision to test was correlated with previous acceptance of a Down syndrome screening in pregnancy, and with lack of worries about emotional, employment, and insurance discrimination (Quinlivan et al.). A fear of discrimination is among the biggest problems in the decision-making process.

The American College of Medical Genetics and Genomics (ACMG) has recommended analysis of 56 specific genes when reporting incidental or secondary findings from next-generation sequencing (NGS) technologies (2). Germline mutations in 16 of these genes cause hereditary cancer syndromes. The ACMG later updated its recommendations, based on the general consensus that patients should be able to opt out of the analysis of secondary findings, during the pre-test counseling sessions. Some of these cancer syndromes may have pretty early onset, so these guidelines are also applied to children, whose parents shall have to make the decision of opting out or not (3). An elegant recent review by Kuhlen and Borkhardt shows that following the recommendations of national and international human genetic societies and the legislation of most European countries, children and their parents must be previously and thoroughly informed about which findings should be reported through a written consent. The ordering physician is obliged to discuss with the child and/or parents all the possibilities of results, including the disclosure of incidental findings, the “right not to know,” the benefits and risks, as well is who has the responsibility for obtaining the informed consent and providing pre- and post-test counseling (4).

The clinical utility and validity of using NGS for hereditary cancer risk assessment are becoming a reality in cancer genetics clinics. One advantage of NGS is the feasibility of including multiple genes in panels tailored to a certain familial aggregation of tumors, such as breast or colon cancer. Nevertheless, because of its economic viability, whole-exome/genome sequencing has displaced the phenotype-driven hypothesis approach that is based on the characteristics and genotype–phenotype correlations of the syndrome. Slavin et al. showed interesting results about multigene panels in a reference cancer genetics clinic. When panels included only high-risk genes, the results were seldom positive, and more variants of unknown significance (VUS) were revealed. This result is likely because of the inclusion of more genes in these so-called “off-phenotype” pan-cancer panels (Slavin et al.). Compared to single-gene tests, cancer panels are time- and cost-efficient in cases with (1) substantial genetic or locus heterogeneity, (2) high prevalence of actionable mutations in one of several genes, (3) difficulty in predicting the mutated gene based on phenotype or family history alone, or (4) non-informative or unavailable family history (e.g., adoption) (5). Recent evidence-based guidelines, such as those of the National Comprehensive Cancer Network, have included the use of multigene testing for familial breast and ovarian cancer risk assessment, comparing its advantages and disadvantages (6).

A critical disadvantage of NGS is the possibility of disclosing inconclusive or uncertain results. Identification of a VUS hampers the interpretation of phenotype and genotype data, rendering genetic counseling a stressful task. As Slavin et al. stated, choosing a phenotype-specific panel with genes of high clinical utility instead of pan-cancer panels with many low-risk genes can decrease the chance of finding variants that are difficult to interpret (Slavin et al.). Pinheiro et al. discussed these issues, offering contrasting arguments. They showed that there is now a tendency for relaxing clinical criteria to select for genetic testing those individuals in a family with the highest risk of cancer, such as individuals with a very early age of onset with no affected relatives. A specific syndrome and specific gene would no longer need to be targeted for testing if a multigene panel were available. However, the value of a strategy with less stringent criteria may be debatable because of the costs of screening, as more individuals would become eligible for testing, despite the costs of NGS continuing to decline (Pinheiro et al.).

Therefore, there must be a rationale to offer the proper genetic test to a family member with cancer. There is an urgent need for implementing multidisciplinary teams, led by a cancer geneticist, in reference settings. Such teams will provide patients and their relatives with the best-informed decision-making process and risk assessment, as we balance the breakthroughs and challenges of the NGS era.

## Author Contributions

The author confirms being the sole contributor of this work and approved it for publication.

## Conflict of Interest Statement

The author declares that the research was conducted in the absence of any commercial or financial relationships that could be construed as a potential conflict of interest.
